# Basal glucose excretion in dogs: The impact of feeding, obesity, sex, and age

**DOI:** 10.1111/vcp.12899

**Published:** 2020-09-24

**Authors:** Florian K. Zeugswetter, Ilse Schwendenwein

**Affiliations:** ^1^ Clinical Department for Small Animals and Horses University of Veterinary Medicine Vienna Vienna Austria; ^2^ Department of Pathobiology Central Laboratory University of Veterinary Medicine Vienna Vienna Austria

**Keywords:** canine, quantitative urine glucose measurement, urine dipstick, urine glucose, urine glucose‐to‐creatinine ratio

## Abstract

**Background:**

The urine glucose (UG) measurements are an integral part of urinalyses, especially in dogs with polyuria and polydipsia. A positive dipstick result is considered pathologic for disease. This paradigm has been challenged by new ultrasensitive tests, where the manufacturers recommend tolerating slightly positive results. It implies that, as in other species, basal urine glucose losses can exceed the lower limits of detection using ultrasensitive glucose dipsticks in healthy dogs.

**Objectives:**

We aimed to determine whether glucose is routinely detectable using a sensitive quantitative wet chemistry method in the urine of nondiabetic, nonazotemic dogs, and investigate the impact of food intake, obesity, sex, castration status, and age.

**Methods:**

Serial UG measurements were performed in healthy clinic‐owned Beagle dogs that were randomly fasted or fed. Glucose was measured in morning urine samples from normal‐weight healthy and obese dogs, and the university's electronic database was searched for quantitative UG measurements (Gluco‐quant Enzyme Kit/Roche Diagnostics).

**Results:**

Small amounts of glucose were detected in 555 (99.1%) of 560 urine samples analyzed. All urine samples from the clinic‐owned Beagle dogs, as well as from privately owned obese and normal‐weight healthy dogs that tested positive for glucose. The median (range) UG concentration obtained from the university's electronic database was 0.39 (0‐1.55) mmol/L, and 2.2% of the samples tested negative. Feeding, obesity, gender, castration status, and age did not affect UG concentrations.

**Conclusions:**

Studies, including a larger number of healthy dogs, are warranted to define a cut‐off between physiologic and pathologic glucosuria.

## 1 INTRODUCTION

Semiquantitative urine glucose (UG) measurements are an integral part of urinalyses in dogs. Documented causes of glucosuria in dogs include diabetes mellitus (DM), hyperglycemia caused by α2 receptor agonists,^1^ renal glucosuria,^2^ Fanconi syndrome,^3^, ^4^, ^5^, ^6^ acute and chronic renal failure,^7^ leptospirosis,^8^ lead toxicity,^9^ and the application of sodium‐glucose cotransporter 2 inhibitors.^10^

False‐positive dry reagent strip results (“pseudoglucosuria”) can be caused by antibiotics such as cephalexin,^11^ contamination by disinfectants such as hydrogen peroxide,^12^ and prolonged exposure of the reagent strips to air.^13^ It is noteworthy that experimental blood contamination caused by adding euglycemic blood samples onto strips had no significant effect on UG scores.^14^ The use of automated dipstick readers can reduce the error rates associated with dipstick urinalyses.^15^ A recent study found discordant results between urine dip and urine drip method, with more trace positive glucose results in the urine of nondiabetic dogs without evidence of tubular disease when using the drip method.^16^

The lowest UG concentration estimate using traditional test strips was 2.8 mmol/L (50 mg/dL, eg, Combur 9; Roche Diagnostics) or 5.5 mmol/L (100 mg/dL, eg, Multistix; Bayer), and a positive result is considered pathologic for disease. Uncertainty exists on the interpretation of results obtained by new ultrasensitive test strips, such as the Medi‐Test Combi 10 VET (Machery Nagel) with an initial point estimate of 1.1 mmol/L (20 mg/dL). Although the manufacturers state that canine urine usually tests negative, they also recommend classifying slight positive results as normal.

The objectives of this study were to determine whether basal glucose excretion is habitually detectable in the urine of nondiabetic dogs when analyzed by a sensitive automated wet chemistry method. The scientific background was, that urine of humans and cats is rarely free of glucose, and glucose concentrations up to 1.4 mmol/L [25 mg/dL] and 1.5 mmol/L [26.7 mg/dL], respectively, are considered physiologic^12^, ^17^, ^18^ Additionally the effects of possible influencing factors, including food intake, obesity, sex, and age were investigated.

## 2 MATERIALS AND METHODS

### 2.1 Study design

This study consisted of three parts, of which two were prospective (part 1 and 2), and one was retrospective (part 3). It included urine glucose measurements from healthy clinic‐owned Beagle dogs (part 1), privately owned healthy normal‐weight and obese dogs (part 2), and urine samples submitted to the University of Veterinary Medicine Vienna laboratory by a local practitioner (part 3). The study was approved by the institutional ethics and animal welfare committee in accordance with good scientific practice (GSP) guidelines and national legislation (10/12/97/2014 and ETK 09/03/2015).

### 2.2 Impact of Feeding

Eight clinic‐owned Beagles older than 1 year and housed in indoor‐outdoor runs were enrolled. There were six castrated and two intact male dogs from 2 years to 5 years (median 3 years) with a body condition score (BCS) of 5‐7 of 9 (median 5). The body weights ranged from 14.4 to 21.6 kg (median 19.1 kg). Medical histories and physical examinations revealed no evidence of disease.

The experiment was performed over 4 days in familiar surroundings. On the first day, the dogs were accustomed to the handling procedures and were assigned to one of two groups (A and B) by simple block randomization using a shuffled deck of cards. Assignment to group A or B defined the sequence of meal/no meal or no meal/meal on days 2 and 3. To test for reproducibility, all dogs received an additional meal on day 4. Urine collection started at 8:00 AM and ended at 1:30 PM. The meal, given at 9:30 AM (the routine feeding time, one meal per day), consisted of a standard dry food (Sensitivity control, Royal Canin, Bruck an der Leitha, Austria) 10g/kg body mass mixed with 12 grams boiled turkey/kg body mass. Water was available ad libitum. The aim was to obtain 12 free‐catch urine samples (samples 1‐4 = prefeeding time, samples 5‐12 = postfeeding time) collected every 30 minutes for 5.5 hours.

### 2.3 Impact of obesity

Owners of 269 dogs aged 1 to 11 years, acquired via social media, public bulletins, or personal contact, who considered their dogs to be of normal weight (BCS 4/9) or obese (BCS 8 or 9/9), but otherwise healthy, were asked to fill out a questionnaire. To exclude dogs with diseases affecting urine glucose concentrations, the questionnaire included inquiries about the eating and drinking habits, orthopedic or endocrine problems, gastrointestinal and respiratory signs, recent stressful events, medications, and neoplasia. The return rate was 54% (n = 146), and 40 dogs were excluded. From the questionnaire, the reasons for exclusion were medications (n = 9), neoplasia, or a history of malignant tumors (n = 8), cardiorespiratory signs (n = 7), polyuria/polydipsia (n = 6), age (n = 5), recent stressful events (n = 3), skin alterations (n = 3), lethargy (n = 3), gastrointestinal signs (n = 2), liver disease (n = 1), bladder stones (n = 1), the BCS was too low (n = 1), and spinal problems (n = 1). Thirty‐eight dogs left the study because owners were unable or unwilling to collect urine samples. Clinical examinations and urinalyses excluded further 19 and 4 dogs, respectively. From the clinical examinations and urinalyses, the reasons for exclusion were higher or lower BCSs (n = 9), neoplasia (n = 7), brachycephalic syndrome (n = 2), generalized lymphadenopathy (n = 1), reddened mucous membranes (n = 1), turbid urine (n = 2), active urine sediment (n = 2), and alkaline urine (n = 1). The study population finally consisted of 25 normal weight and 20 obese dogs.

### 2.4 Impact of sex and age

The electronic database (TIS VetWare, Agfa HealthCare, Vienna, Austria) of the central laboratory (University of Veterinary Medicine Vienna/Austria) was searched for quantitative urine glucose measurement results in canine urine performed between 2007 and 2017. The search terms applied were “dogs” and “urine glucose.” In the case of multiple consignments from the same animal, only the results of the first urine sample were included. Dogs with DM (plasma glucose >8 mmol/L [144 mg/dL]^19^ and glucosuria [dipstick] and/or plasma fructosamine >370 µmol/L), isolated hyperfructosaminemia, acute or chronic kidney disease (plasma creatinine >1.6 mg/dL and urine specific gravity (USG) <1.030),^7^ and known thyroid, glucocorticoid,^20^ or chemotherapeutic treatments were excluded. Of 622 urine glucose measurements, 355 (multiple measurements), 28 (kidney disease), 14 (DM, three of these also had kidney disease) and, one sample (hyperfructosaminemia) were excluded. The final study population consisted of 227 dogs.

### 2.5 Urinalysis

From samples obtained in Groups 1 and 2, aliquots of freshly voided urine were transferred from a special collection device to microtubes (Vacuette; Greiner bio‐one), and centrifuged at 1500 rpm and 10 000 rpm for 3 minutes for sediment analysis and storage of supernatant, respectively. To avoid microbial metabolism, samples were frozen at −80°C within 2 h. Urine samples from Group 3 were processed as described above within 2‐4 hours without freezing. All samples underwent a conventional urinalysis including the determination of urine specific gravity (USG) by refractometry (daily calibration), microscopic sediment evaluation, and semiquantitative multi‐dipstick analysis (Combur 9 Test; Roche Diagnostics). Quantitative determinations of urine UG and creatinine concentrations were performed on a fully selective chemistry analyzer (Cobas c 501; Roche Diagnostics) with the hexokinase (Gluco‐quant Enzyme Kit/Roche Diagnostics) and creatininase methods (Creatinine plus version 2; Roche Diagnostics), respectively. The linearity range claimed by the manufacturer for UG was 0.11‐41.6 mmol/L (1.8‐749 mg/dL). A dilution/recovery study was performed using canine urine with high (78 mmol/L [1402 mg/dL]) and unmeasurable glucose concentrations. The intercept and slope were 14.72 and 0.989, respectively, whereas the average recovery was 111%. The intra‐assay coefficients of variation were calculated by assessing 10 replicates of urine samples (mean ± SD) with low (4.1 ± 0.04 mmol/L [73 ± 0.7 mg/dL]), medium (15.5 ± 0.2 mmol/L [280 ± 4 mg/dL]), and high (31.2 ± 0.6 mmol/L [563 ± 11.6 mg/dL]) glucose concentrations. The coefficients of variation were 2.1, 1.4, and 0.9%, respectively. Day to day precision was determined by measuring two commercially available aqueous control solutions (Lyphocheck 1 and 2, Biorad, Vienna; target values 1.2 and 17 mmol/L) over 10 consecutive days, which revealed CV%s of 5 and 1.8%, respectively. These control solutions were also used for daily internal quality control checks. Samples with analyte concentrations exceeding the linearity range were automatically diluted 1:20 and reanalyzed. Turbid samples, urine proteins> +, or hematuria> (+) were excluded from the analyses. Finally, data from 560 quantitative and 544 dipstick urine glucose measurements were included.

### 2.6 Statistics

Statistical analyses were performed with the laboratory software package IBM SPSS Statistics 24. Normal distributions of data were tested with the Kolmogorov‐Smirnov test. If data were not normally distributed, nonparametric tests were applied. The differences between groups (male vs female dogs and obese vs normal‐weight dogs) were analyzed using the Mann‐Whitney test. In the case of dependent variables (impact of feeding), the Wilcoxon signed‐rank test was applied. Post‐hoc Bonferroni‐Holm corrections were used in part 1 of the study (impact of feeding) to account for multiple comparisons. To avoid gender bias in part 2 of the study, male and female distributions were compared using the chi‐square test. Correlations between UG and other parameters (eg, age, weight, plasma glucose, urine protein, urine specific gravity) were tested using the nonparametric Spearman's rank correlation coefficient. After excluding outliers, defined as data higher than three interquartile ranges (3 IQR) above the third quartile (Q3), the 0.975‐fractiles were calculated. Data are given as the median and range, and the level of significance was set at *P* < .05.

## 3 RESULTS

### 3.1 Impact of feeding

**FIGURE 1:**
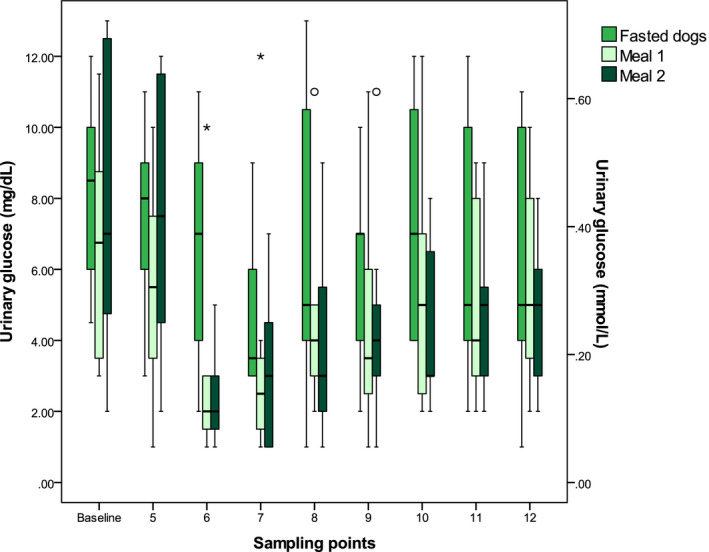
Box and whisker plots of glucose concentrations in free‐catch urine samples from 8 healthy Beagle dogs collected at 12 different sampling points either fasted, or before (baseline) and after feeding (sampling points 5‐12) The boxes represent the 25th and 75th percentiles, and the horizontal line indicates the median value. The whiskers indicate the range of values below and above the first (Q1) and third (Q3) quartiles up to 1.5 times the interquartile range, respectively. Values lying more than 1.5 or 3 times below Q1 or above Q3 are outliers and depicted as dots or stars, respectively

All dogs ate the meals within 5 minutes, and the collection of adequate urine samples was possible at most sampling points. When fasted, urine production decreased, and urine collection was not possible at every sampling point. Eggs of *Capillaria plica* were detected in the urine sediment of four dogs. These dogs showed no signs of inflammation, for example, hematuria or active sediments. Feeding did not affect UG concentrations or the UGCRs (Figure 1; Table 1). Glucose was detectable in all urine samples using wet chemistry analyses, but no dog was glucosuric using the Combur 9 dipstick test.

**TABLE 1:** Urine glucose (UG) concentrations, urine glucose‐to‐creatinine ratios, and urine creatinine (UC) concentrations in fasted or fed Beagles (n = 8) with 12 free‐catch urine samples collected

Urine Sampling times																			
		1‐4			5		6		7		8		9		10		11		12
Urine glucose concentrations (mg/dL)																			
Meal (Day 1)	6.75 (3‐12)		5.5 (1‐10)			2 (1‐10)		2.5 (1‐12)		4 (2‐11)		3.5 (1‐11)		5 (2‐12)		4 (2‐9)		5 (2‐10)	
Meal (Day 2)	7 (2‐13)		5.5 (2‐12)			2 (1‐5)		2.5 (1‐7)		4 (1‐9)		3.5 (1‐11)		5 (2‐8)		4 (2‐9)		5 (2‐8)	
No meal	8.5 (4.5‐12)		8 (3‐11)			7 (2‐11)		3.5 (3‐9)		5 (1‐13)		7 (2‐10)		7 (4‐12)		5 (2‐12)		5 (1‐11)	
Urine glucose concentrations (mmol/L)																			
Meal (Day 1)	0.375 (0.167‐0.666)			0.305 (0.056‐0.555)			0.111 (0.056‐0.555)		0.139 (0.056‐0.666)		0.222 (0.111‐0.611)		0.194 (0.056‐0.611)		0.278 (0.111‐0.666)		0.222 (0.111‐0.5)		0.278 (0.111‐0.555)
Meal (Day 2)	0.389 (0.111‐0.722)			0.305 (0.111‐0.666)			0.111 (0.056‐0.278)		0.139 (0.056‐0.389)		0.222 (0.056‐0.5)		0.194 (0.056‐0.611)		0.278 (0.111‐0.444)		0.222 (0.111‐0.5)		0.278 (0.111‐0.444)
No meal		0.472 (0.25‐0.666)			0.444 (0.167‐0.611)		0.389 (0.111‐0.611)		0.194 (0.167‐0.5)		0.278 (0.056‐0.722)		0.389 (0.111‐0.555)		0.389 (0.222‐0.666)		0.278 (0.111‐0.666)		0.278 (0.056‐0.611)
Urine glucose‐to‐creatinine ratios																			
Meal (Day 1)		0.041 (0.036‐0.050)			0.039 (0.037‐0.047)		0.045 (0.036‐0.072)		0.049 (0.037‐0.062)		0.052 (0.044‐0.053)		0.054 (0.027‐0.064)		0.054 (0.05‐0.06)		0.052 (0.041‐0.067)		0.057 (0.04‐0.066)
Meal (Day 2)		0.04 (0.035‐0.053)			0.041 (0.037‐0.051)		0.045 (0.034‐0.062)		0.045 (0.037‐0.073)		0.057 (0.036‐0.062)		0.051 (0.038‐0.067)		0.053 (0.045‐0.079)		0.057 (0.043‐0.081)		0.056 (0.04‐0.091)
No meal		0.044 (0.032‐0.049)			0.042 (0.033‐0.058)		0.040 (0.032‐0.048)		0.045 (0.031‐0.064)		0.038 (0.030‐0.056)		0.047 (0.036‐0.058)		0.041 (0.036‐0.051)		0.046 (0.031‐0.052)		0.041 (0.036‐0.056)

Samples 1‐4, prefeeding times; samples 5‐12, postfeeding times. Urines were collected every 30 minutes over 5.5 hours. Data given as the median (range). Differences between glucose concentrations and UGCRs measured at the sampling points 1‐4 and 5‐12 are not significant (Bonferroni‐Holm correction was applied).

### 3.2 Impact of obesity

Dogs in the normal‐weight group (n = 25) averaged 4 (1‐11) years of age. There were 11 male (5 intact) and 14 female (2 intact) dogs. Body mass indices averaged 21 (7‐22) kg. There were 14 purebred (8 different breeds) and 11 mixed breed dogs. Nine and 16 dogs had a BCS of 4/9 and 5/ 9, respectively.

The dogs in the obese group (n = 20) averaged 5 (1.5‐10) years of age. There were 7 males (2 intact) and 13 females (4 intact). Body mass indices averaged 15.7 (5‐44) kg. There were 11 purebred (9 different breeds) and 9 mixed breed dogs. Fourteen and 6 dogs had a BCS of 8/9 and 9/ 9, respectively.

There was no difference of age (*P* = .384) or gender (*P* = .54) between the groups.

**FIGURE 2:**
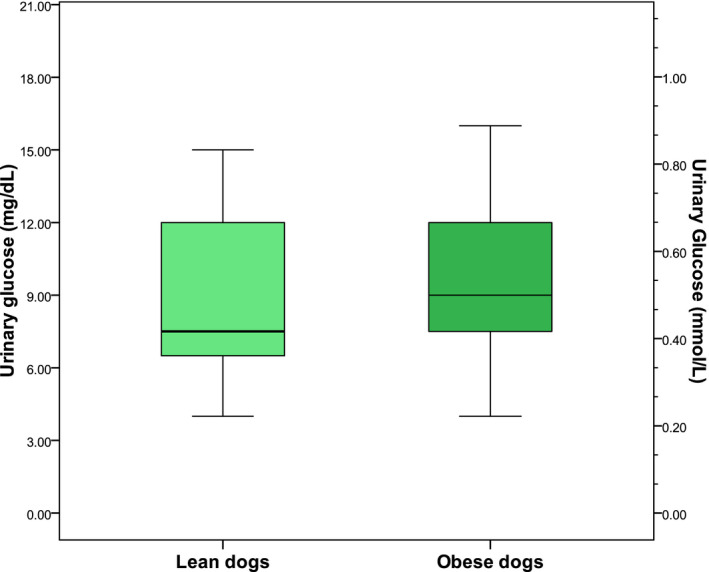
Box and whisker plots of glucose concentrations in urine samples from 25 lean healthy and 20 obese, but otherwise healthy dogs. The boxes represent the 25th and 75th percentiles, and the horizontal line indicates the median value. The whiskers indicate the range of values below and above the first (Q1) and third (Q3) quartiles up to 1.5 times the interquartile range, respectively. Values lying more than 1.5 or 3 times below Q1 or above Q3 were considered outliers and are depicted as dots or stars, respectively. The difference between the groups was not found to be significant (*P* = .21)

UG and the UGCR were comparable between lean and obese dogs (Figure 2; Table 2). Low, but nonsignificant correlations were found between UG and BCS (*r*_SP_ = .178, *P* = .242) and UG and weight (*r*_SP_ = −.198, *P* = .191). UGCR correlated positively with BCS (*r*_SP_ = .320, *P* = .032) and negatively with weight (*r*_SP_ = −.405, *P* = .006). Glucose was detectable in all urine samples, but no dogs were glucosuric using the Combur 9 dipstick test.

**TABLE 2:** Urine glucose (UG) concentrations and the urine glucose‐to‐creatinine ratios (UGCRs) of normal‐weight healthy (n = 25) and obese, otherwise healthy (n = 20) dogs

	Normal‐weight dogs	Obese dogs	*P*‐value
Number of dogs	25	20	
UG (mg/dL)	8 (4‐15)	9 (4‐16)	.21
UG (mmol/L)	0.444 (0.056‐0.611)	0.5 (0.222‐0.888)	.21
UGCR	0.042 (0.024‐0.067)	0.048 (0.027‐0.075)	.278

### 3.3 Impact of sex and age

The 227 dogs in group 3 averaged 9.4 (0.25‐17) years of age. Sixty‐nine males (42 intact) and 103 females (47 intact) were included. Sex was not documented in 55 dogs. There were 126 purebred dogs representing 57 different breeds. The breeds with >3 dogs were Yorkshire Terrier (7), Maltese (7), Standard Poodle (6), Jack Russell Terrier (6), Border Collie (5), Dalmatian (5), Golden Retriever (5), and Weimaraner (4). There were also 42 mixed breed dogs. A breed was not documented in 59 dogs. Most of the urine samples under investigation were submitted by one local veterinarian for quantitative glucose measurements as part of the yearly routine health check or to rule out glucosuria in cases of polyuria/polydipsia. UG was below the detection limit of the assay in 5 (2.2%) dogs. Glucose concentrations were above 1.11 mmol/L (20 mg/dL; all dipstick negative) in 16 (7.1%) dogs and 2.78 mmol/L (50 mg/dL; positive dipstick test in one, no dipstick test performed in two) in 3 (1.3%) dogs. After excluding five outliers (80.3 mmol/L [1447 mg/dL], 8.3 mmol/L [150 mg/dL], 5.4 mmol/L [98 mg/dL], 2.2 mmol/L [39 mg/dL], and 1.9 mmol/L [35 mg/dL]), the median (range) UG concentration was 0.39 (0‐1.55) mmol/L or 7 [0‐28] mg/dL in the 222 remaining dogs. The median (range) UGCR was 0.047 (0‐0.16). The 0.975‐fractiles were 1.3 mmol/L (23.4 mg/dL) for UG and 0.146 for the UGCR.

**FIGURE 3:**
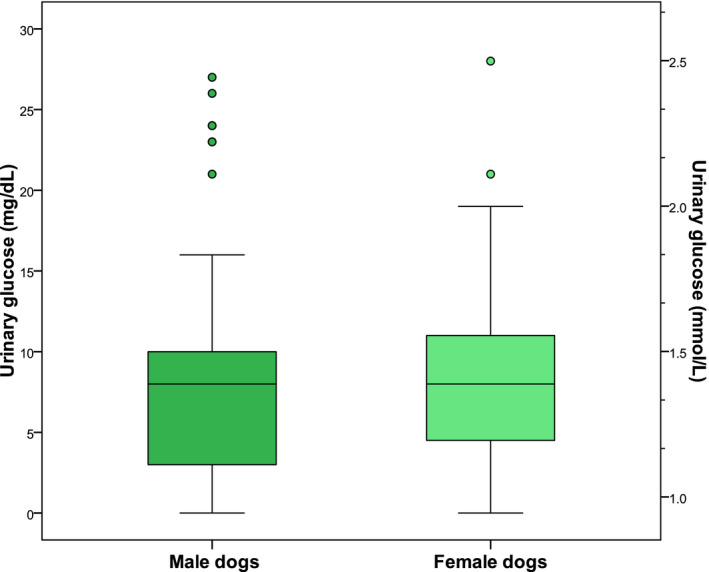
Box and whisker plots of glucose concentrations in urine samples from nondiabetic and nonazotemic male (n = 69) and female dogs (n = 103) submitted by a small animal practitioner. The boxes represent the 25th and 75th percentiles, and the horizontal line indicates the median value. The whiskers indicate the range of values below and above the first (Q1) and third (Q3) quartile up to 1.5 times the interquartile range, respectively. Values lying more than 1.5 or 3 times below Q1 or above Q3 are outliers and depicted as dots or stars, respectively. The difference between the groups was not found to be significant (*P* = .421)

Gender had no effect on UG (male vs female: *P* = .421, Figure 3) or UGCR (male vs female: *P* = .54). Castration status also had no effect of UG (castrated vs intact males, *P* = .767; spayed vs intact females: *P* = .544) or UGCR (castrated vs intact males: *P* = .472; spayed vs intact females: *P* = .291).

UG correlated positively with urine specific gravity (*r* = 0.539, *P* < .001) and urine protein (*r* = 0.416, *P* < .001), but not with age (*r* = −.136, *P* = .061), UPCR (*r* = .01, *P* = .887), or plasma glucose (*r* = .161, *P* = .105). UGCR correlated positively with age (*r* = .189, *P* = .011), urine protein (*r* = .224, *P* < .001), and UPCR (*r* = .291, *P* < .001), but not with plasma glucose (*r* = .091, *P* = .374).

Standard Poodles (*P* = .024) and Yorkshire Terriers (*P* = .012) had higher UG concentrations, whereas Golden Retrievers (*P* = .01) had lower UG concentrations than the other dogs. The UGCR was higher in Yorkshire Terriers (*P* = .018), and lower in Golden Retrievers (*P* = .024).

A urine glucose dipstick test (Combur 9 Test) was performed in 212 (93.4%) dogs and was positive in three (1.4%; results of the chemistry analyzer: 80.3 mmol/L [1447 mg/dL], 0.555 mmol/L [10 mg/dL], and 0.278 mmol/L [5 mg/dL]). The first dog likely had renal glucosuria since the blood glucose concentration was 3.83 mmol/L (69 mg/dL), and the dog was not treated for DM. The cause of the false‐positive test results in the other two dogs could not be identified.

## 4 DISCUSSION

Using a highly sensitive quantitative UG assay, we detected small amounts of glucose in 99.1% of 560 canine urine samples, which contradicts the paradigm, that urine is virtually glucose free provided the renal tubular maximum for glucose reabsorption is not exceeded.^1^ Only five (2.2%) of the 227 urine samples submitted by a local small animal practitioner tested negative for glucose when analyzed by wet chemistry. Negative glucose measurements were not observed in healthy Beagle dogs irrespective of the feeding status, nor were they observed in obese and normal‐weight healthy privately owned dogs. Accordingly, the terms “normoglucuria” or “basal glucosuria” as established in humans^17^, ^18^, ^21^ and suggested for cats,^12^ seem appropriate for use in the canine species. Nevertheless, as in nondiabetic humans^17^and cats,^12^ glucose concentrations measured with laboratory methods were low and rarely above the detection limits of currently available traditional urine glucose dipstick tests, with a first point estimate of 2.8 mmol/L (50 mg/dL). The fact that only 1.4% of our dogs had positive dipstick test results is in stark contrast to an earlier study,^1^ where positive test results were observed in 7.4% of dogs of other breeds than Norwegian Elkhounds (breed with known familial kidney disease) during dog shows in Norway. As all of these dogs were clinically healthy and drinking patterns were normal, the authors suggested longstanding stress‐related hyperglycemia with associated glucosuria or less likely methodologic errors related to the test strips as possible causes and recommended studies using quantitative laboratory urinalysis.

Glucose concentrations were above 1.1 mmol/L (20 mg/dL) in 7.1% of the urine samples from nondiabetic dogs examined in this study. The 0.975‐fractile of all measurements was 1.3 mmol/L (23.4 mg/dL). As some dogs under investigation possibly suffered from proximal renal tubular dysfunction, further research including strictly controlled healthy dogs is encouraged to calculate upper reference limits to clarify whether urine glucose concentrations ≥1.1 mmol/L (20 mg/dL) are normal in dogs. This is of interest as manufacturers of recently launched, highly sensitive veterinary test strips with a first point estimate at 1.1 mmol/L (20 mg/dL), state that slightly positive glucose reactions are possible in healthy dogs. Moreover, glucose concentrations never exceeded 0.72 mmol/L (13 mg/dL) in our healthy Beagle dogs, irrespective of feeding status. These concentrations also did not exceed 0.89 mmol/L (16 mg/dL) in the 20 obese but otherwise healthy dogs.

As shown in eight healthy clinic‐owned Beagle dogs, with multiple measurements over 5.5 hours, UG variability was small, and food intake had no significant short‐term effects. The lack of UG variability and food effects was unexpected. Glucose reabsorption and consequently, glucose excretion is regulated mainly by sodium‐glucose cotransporters 2 (SGLT 2) located at the apical membrane of renal proximal tubular cells. ^22^ These low affinity but high capacity glucose transporters are insulin sensitive and reabsorb about 90% of glucose together with insulin‐independent basolateral GLUT2 transporters under normal conditions.^22^ The elimination of insulin receptors expressed on renal tubular cells,^23^ reduces SGLT 2 expression and increases UG excretion.^24^ Human patients with increased insulin concentrations are more likely to have low UG concentrations independent of blood glucose concentrations.^25^ As insulin increases postprandially, and peak blood glucose concentrations do not exceed the so‐called “renal threshold” in healthy dogs,^26^ a drop in postprandial UG concentrations was expected. Although median UG concentrations dropped after meal intake, the differences did not reach statistical significance. The plasma glucose concentration above which SGLT capacities are saturated and pathologic glucosuria begins is defined as the renal threshold.

Glucose concentrations in the urine of obese but otherwise healthy nondiabetic dogs were comparable to the concentrations measured in lean dogs. The rationale for comparing UG concentrations between lean and obese dogs was twofold. Obesity causes significant structural, biochemical, hemodynamic, and functional changes in the canine kidneys,^25^ possibly impairing glucose handling. Secondly, although dogs are protected from type 2 diabetes mellitus even after years of naturally occurring obesity, they can develop obesity‐induced insulin resistance and compensatory hyperinsulinemia.^27^, ^28^ We hypothesized that obesity‐associated tubular damage, or a combination of insulin resistance and hyperinsulinemia, would affect SGLT‐2 dependent glucose reabsorption and consequently, UG excretion. In a recently published Japanese study including 184, 160 non‐DM people receiving general health screenings, pathologic glucosuria was detected in 470 (0.26%) of the participants and tended to be associated with increased body mass indices, waist circumferences, and serum creatinine concentrations.^29^ In a study of obese diabetic fatty rats, UG concentrations increased 10 weeks before blood glucose levels exceeded renal thresholds, which was after the rats had received 12 weeks of a high‐fat diet that nearly doubled their body masses.^30^

As in cats,^12^ no significant sex or castration status effects were observed. This was of interest as the expression of the SGLT‐2s exhibits sex and species differences. The SGLT‐2 protein shows higher expression in female rats than male rats. It is enhanced by estradiol and downregulated by androgens. Interestingly, the increased expression in female rats was not accompanied by higher SGLT‐2‐dependent glucose uptake on brush‐border vesicles compared with male rats, suggesting a functional contribution of another glucose transporter system. In contrast to the rat kidneys, the SGLT‐2 protein is dominant in male mice.^31^ In a human study, including 261 healthy volunteers, no gender differences were found between males and females for up to 50 years, but women above this age had significantly lower mean urine glucose concentrations.^32^ In a large Japanese study including participants with a mean (± SD) age of 63.5 (± 8.4) years, only 28.3% of the non‐DM subjects with pathologic glucosuria were female.^29^

In contrast to cats,^12^ no correlations were found between the ages and UGs in the dogs of this study. Human studies that included healthy subjects^33^ or type‐2 diabetic patients^34^ demonstrated rising renal glucose thresholds with age. Accordingly, higher blood glucose concentrations are needed in aged humans to override the renal capacity to reabsorb glucose. It is possible that our study was underpowered to show an age effect.

Although the data suggest breed differences, the number of dogs in each group was too small to allow reliable conclusions. Norwegian Elkhounds^1^ or Basenjis,^3^ two breeds with known predispositions for renal glucosuria were not included in the study population.

The determination of the UG concentrations does not incorporate urine flow rates, which are modulated by hydration status and renal reabsorption of free water. To allow for dilutional effects, we integrated urine creatinine measurements and calculated the UGCR. Although this is already standard practice for other urine solutes such as proteins^35^ and corticoids,^36^ the authors found only one canine study where the UGCR was calculated to document glucosuria. Five healthy Beagles were artificially made glucosuric by sequentially increasing constant rate glucose infusions. Significant increases in the UGCRs above baseline were observed at serum glucose concentrations of 10‐11.1 mmol/L (180‐200 mg/dL).^37^ In this study, food intake, sex, and castration status did not affect the UGCR; however, the UGCR was positively correlated with age and BCS. Thus, relative age and BCS‐dependent increases in glucose vs creatinine concentrations have to be considered when interpreting UGCRs.

In conclusion, our study results suggest that small amounts of glucose are continually present in canine urine and that glucose excretion is unaffected by the feeding status, obesity, age, or gender. Prospective studies, including a larger group of healthy dogs, are encouraged to define cut‐off values between physiologic and pathologic glucosuria.

## DISCLOSURE

The authors have indicated that they have no affiliations or financial involvement with any organization or entity with a financial interest in, or in financial competition with, the subject matter or material discussed in the article.
